# Static magnetic fields reduce epileptiform activity in anesthetized rat and monkey

**DOI:** 10.1038/s41598-018-33808-x

**Published:** 2018-10-30

**Authors:** Casto Rivadulla, Juan Aguilar, Marcos Coletti, Jordi Aguila, Sandra Prieto, Javier Cudeiro

**Affiliations:** 1 NEUROcom, School of Health Sciences University of A Coruna, and Agrupación estratégica CICA-INIBIC - UdC, A Coruna, Spain; 2 Cerebral Stimulation Centre of Galicia, A Coruna, Spain; 3 Laboratorio de Neurofisiología Experimental, Hospital Nacional de Parapléjicos, Servicio de Salud de Castilla-La Mancha, Toledo, Spain

## Abstract

Increasing evidence indicates that static magnetic fields (SMF) reduce cortical activity in both human and animal models. The aim of this work was to investigate the effect of SMF on epileptiform cortical activity, a condition related to an abnormal increase in neuronal excitability. The first experimental block included a Pilocarpine rat model of epilepsy, in which a magnetic neodymium nickel-plated cylinder, a magnetic field of 0.5 T, or “*sham*” were placed over the skull. In the second experimental block, we recorded epileptic-like activity in the visual cortex of a monkey (Macaca mulatta) under control conditions and in the presence of the magnet. Between 15 and 30 minutes after the second dose of Pilocarpine, EEG changes compatible with seizure like events induced by Pilocarpine were clearly observed in the control animals (sham stimulation). Similar effects were visible in the animals exposed to the real magnet after 1–2 hours. In the monkey, SMF over the cortical focus clearly reduced abnormal activity: the intensity threshold for visual induction increased and the severity and duration decreased. These results reinforce the view that static magnets modulate cortical activity and open the door to the future therapeutic use of SMF in epilepsy as a complement to current pharmacological treatments.

## Introduction

In addition to the development of new drugs, there is an increasing interest in the search for new therapeutic alternatives that enable improvements in the quality of life of patients who suffer from epilepsy, particularly those in which conventional pharmacology is not effective. In these patients, surgery and deep brain stimulation might be viable treatment options^1–3^. In parallel, the research and development of new neuromodulatory tools, such as transcranial magnetic stimulation (TMS), transcranial direct current stimulation (tDCS), focused ultrasound or the use of static magnetic fields, are of substantial importance^4,5^. The actual causes of epilepsy include trauma, vascular accidents, brain tumors and numerous unknown factors; however, from a mechanistic point of view, seizures are the result of a misbalance between inhibition and excitation at the cerebral cortex that results in an increase in the cortical excitability and the consequent formation of ictiogenic networks^6,7^. Cortical excitability appears to be increased in epileptic patients and could be a predictive factor of illness evolution^8^. This is where the emerging non-invasive neuromodulatory techniques previously described, which can decrease cortical excitability, might play a role. Moreover, TMS and tDCS have been used for epilepsy treatments with mixed results, probably due to the use of different stimulation protocols and the different etiologies of the patients^9^.

The application of moderate static magnetic fields (SMF), an alternative neuromodulatory technique, has also demonstrated interesting properties for potential use in epilepsy treatment. Application of SMF at a moderate intensity produces a clear and reproducible decrement of cortical excitability in both human and animal models^10–13^. Furthermore, SMF have the advantage of being cheaper, portable and easier to use compared with the other neuromodulatory techniques. In the specific field of epilepsy, the use of SMF has not often been applied; however, the results obtained are promising, and it has been shown that SMF increase the efficacy of conventional anticonvulsant drugs in black Swiss mice^4,14^.

In this paper, we intend to extend our knowledge regarding the putative usefulness of static magnetic fields in epilepsy. Here, we investigated the effect of 0.5 T neodymium magnets on a pharmacological model of epilepsy in the rat (the lithium-Pilocarpine model) and spontaneously recorded seizures in a primate. In both cases, the abnormal epileptiform activity recorded from the EEG was clearly attenuated. The results reinforce the view that SMF reduce the cortical excitability and suggest that SMF could be considered to be an epileptic adjuvant treatment in the future.

## Results

### Experiment I (rat): 14 animals (7 magnet and 7 sham) were included in this experimental block

#### Static magnetic fields delay the appearance of the seizures induced by Pilocarpine

**Figure 1:**
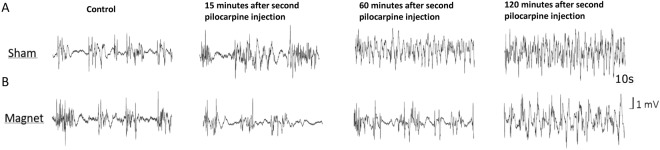
Static magnetic fields delay the appearance of EEG epileptiform activity induced by Pilocarpine. Raw traces (10 sec) from two animals recorded simultaneously under different experimental conditions. (**A**) Sham animal. (**B**) Magnet animal.

Figure 1 shows representative EEG traces from two animals, sham in A and magnet in B. Note that the magnet or sham is placed at the first Pilocarpine dose; thus, there is no magnet in the control recording. In the sham animal, 15 minutes after the second dose of pilocarpine, a clear disorganization of the EEG activity was identified, up and down states (i.e., periods of high and low EEG activity characteristic of anaesthetized conditions) were difficult to differentiate, and there was a tendency towards an oscillatory activity of approximately 4 Hz; none of these effects were visible in the animal with the magnet that continued to exhibit activity similar to the control. One hour after the second dose of pilocarpine, the sham rat showed activity that was compatible with an epileptic status, while the rat with the magnet maintained (somehow) a normal activity, even when an increase in the occurrence of the up states was evident. Finally, 2 hours after pilocarpine, the animal with the magnet also showed a deteriorated EEG compared to the control situation.

#### Static magnetic fields preserve EEG low frequencies induced by anesthesia

**Figure 2:**
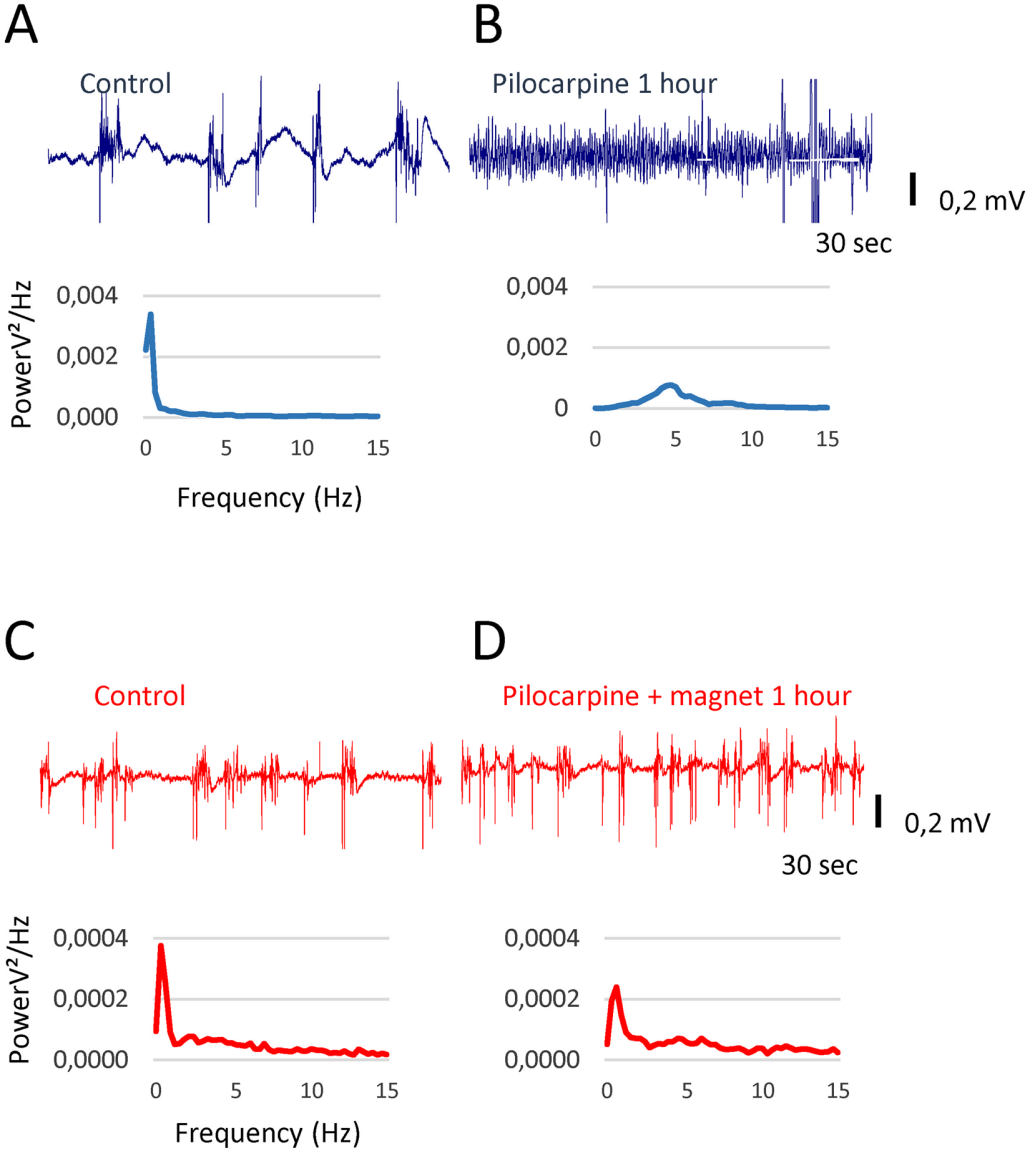
Static magnetic fields delay the development of EEG abnormalities after a Pilocarpine injection and slow down the shift towards higher frequencies. (**A**) Raw EEG recording from the sham animal in the control. (**B**) EEG from the same animal 1 hour after pilocarpine injection. (**C**,**D**) Raw EEG recordings from the Magnet animal under the same experimental conditions as in A and B. Histograms below each recording show the frequency analysis of the signal.

Representative EEG traces from a second pair of animals are shown in Fig. 2 (sham in blue and real stimulation in red). In the control conditions (Fig. 2A,C), both animals had similar up and down states, and the Power Spectra Density (PSD) (curves below the EEG traces) showed a predominance of low frequencies that is typical of the anaesthetized state. One hour after Pilocarpine application in the animal with the magnet (Fig. 2D), an increment of up periods relative to down states was identified; however, the presence of both states remained evident, and the PSD showed that the dominant frequencies remained below 2 Hz. Moreover, the sham (Fig. 2B) showed all of the signs of epileptiform activity, including a reduction in the amplitude of the recording, a continuous up state and a displacement in the power spectrum toward 5 Hz.

**Figure 3:**
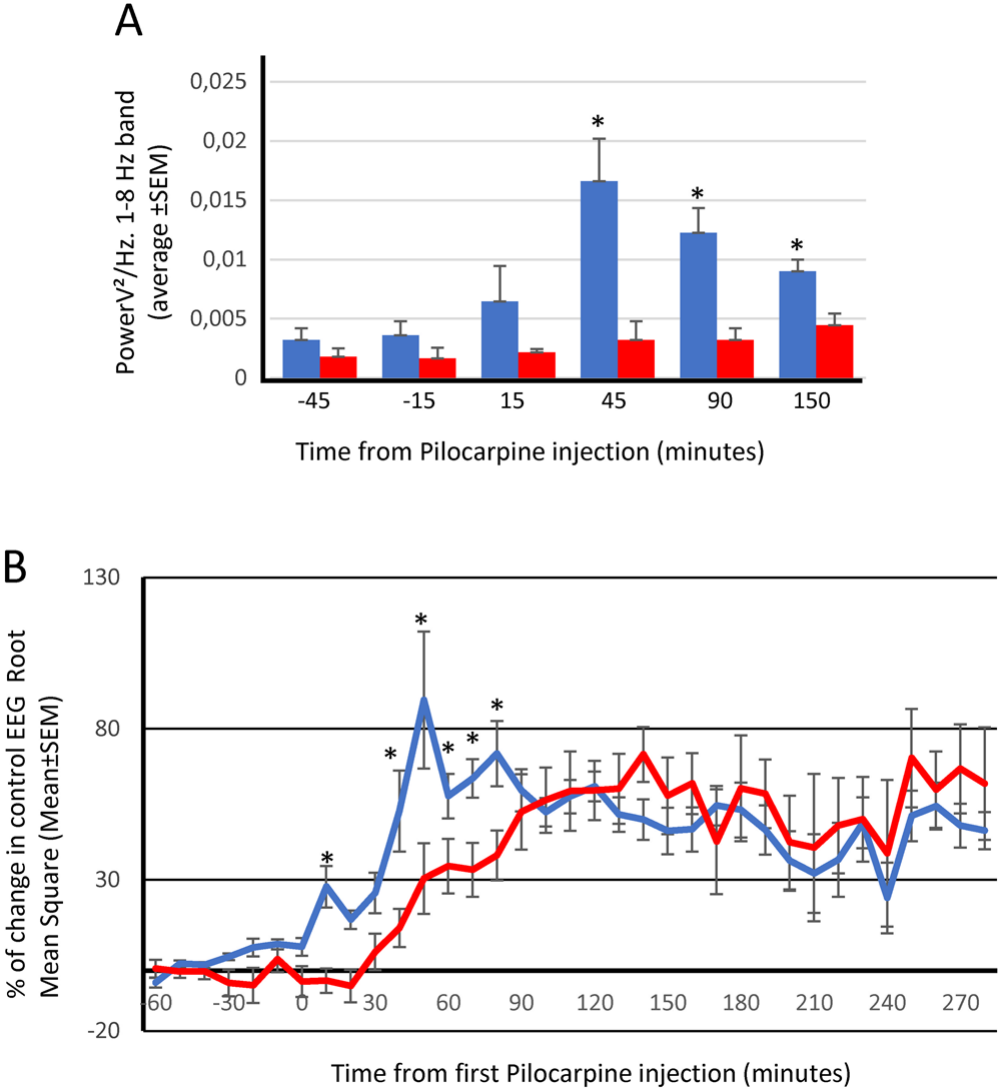
Static magnetic fields prevent changes in frequency and amplitude after a Pilocarpine injection (**A**) Bar histogram representing the averaged potency of the EEG at 1–8 Hz band (PSD) for sham animals (blue bars) and Magnet animals (red bars) ^*^p < 0.05, Mann-Whitney U test. (**B**) Percent change in the spontaneous EEG Root Mean Square (RMS). Blue line, sham rats; red line, Magnet rats. ^*^p < 0.05, Mann-Whitney U test. In both cases (**A**,**B**), data were obtained from 30 second epochs, automatically collected from the original recording every 10 minutes, and actual values are presented in Supplementary Tables S1, S2.

We estimated that Pilocarpine was producing its full effect at the cortical level when the down states were not present in the recording. This time was an average of 40 minutes for the sham animals versus 82 minutes for the rats with the magnet. This finding represents a doubling of the time to the onset of epileptic status (P < 0.05, Mann-Whitney, non-parametric test). Furthermore, as shown in Fig. 3 (and subsequently discussion), where the temporal evolution of the Pilocarpine effect is illustrated, even after long periods following the Pilocarpine injections, the animal with the magnet retained some resemblance to the control situation, which indicates that the magnet not only delays the induction but also decreases the seizure intensity.

Figure 3 shows the temporal evolution of the Pilocarpine effect on the frequencies included between 1 to 8 Hz in the Sham and Magnet animals and on the Root Mean Square of the spontaneous EEG activity (data are averaged values from 7 animals, mean ± SEM). There is an evident displacement in time of both parameters, with pilocarpine-induced changes occurring substantially earlier in the Sham animals than in the Magnet animals, which confirms a protective effect of the magnet.

#### Static magnetic fields preserve sensory responses

**Figure 4:**
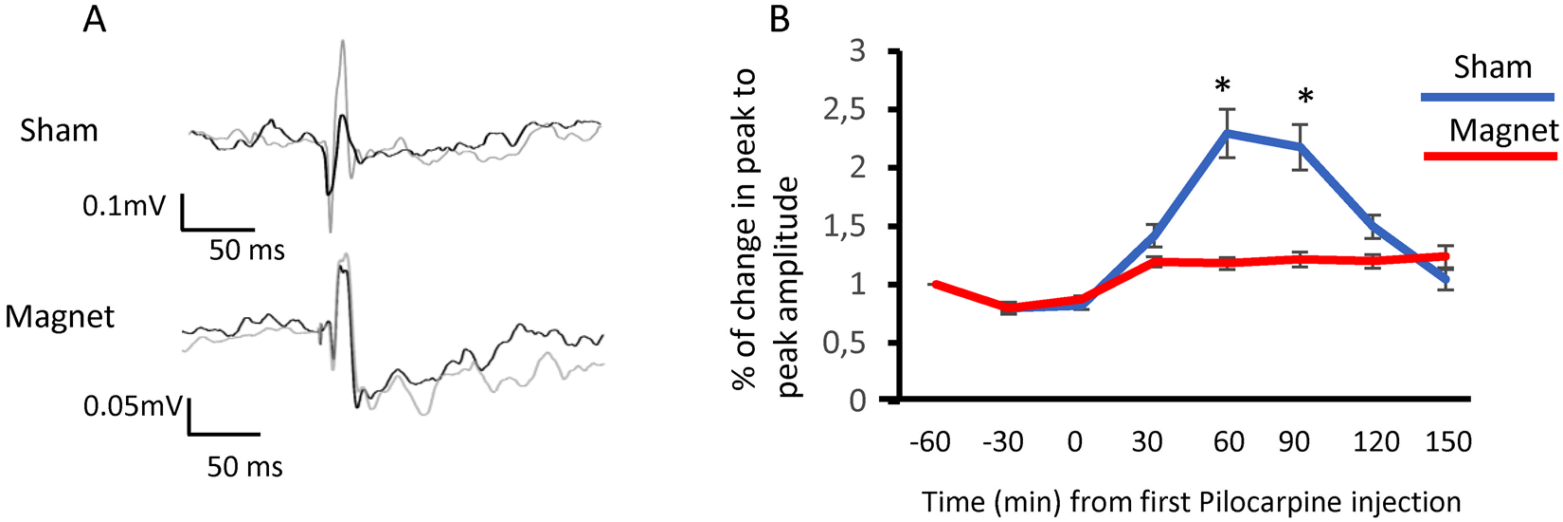
Static Magnetic Fields preserve sensory responses. (**A**) Somatosensory evoked potentials in control (black) and 60 minutes after 1^st^ Pilocarpine injection (grey) in sham (top) and Magnet (bottom) animals. Evoked potentials were averaged over 50 trials. (**B**) Percent change in the peak-to-peak amplitude on the somatosensory evoked potentials, averaged for all animals (7 sham and 7 magnet) after a Pilocarpine injection. Paired sample t-test, ^*^p < 0.05. Actual values are presented in Supplementary Table S3.

Our results suggested that while the cortical excitability was increased in the presence of Pilocarpine in the sham animals, it was reduced in the magnet animals. Thus, this relationship should be reflected in the cortical responses to sensory stimulation. To test this hypothesis, we electrically stimulated the hindpaw at 0.5 Hz and recorded the evoked responses at S1. Figure 4 summarizes the results of this experiment. The panel in A shows the averaged evoked potential in two animals (Sham and Magnet). It is evident that 60 minutes after the 1^st^ dose of pilocarpine, in the recording obtained from the rat with the magnet, the amplitude of the evoked potential (from peak to peak) is similar to the previous amplitude (no significant difference); however, in the Sham animal, there was an increment of 82.2% (p < 0.05 t-test of paired samples). The panel in B shows the temporal evolution of the normalized amplitude of evoked potentials for the whole population (14 rats; 7 rats with real stimulation and 7 rats with sham) after Pilocarpine injection. The magnet was able to maintain a stable sensory response, while in the animals under sham stimulation, the amplitude of the evoked response doubled the control response.

### Experiment II (monkey):1 animal was included in this experimental block

#### Static magnetic fields reduce spontaneous epileptiform activity

**Figure 5:**
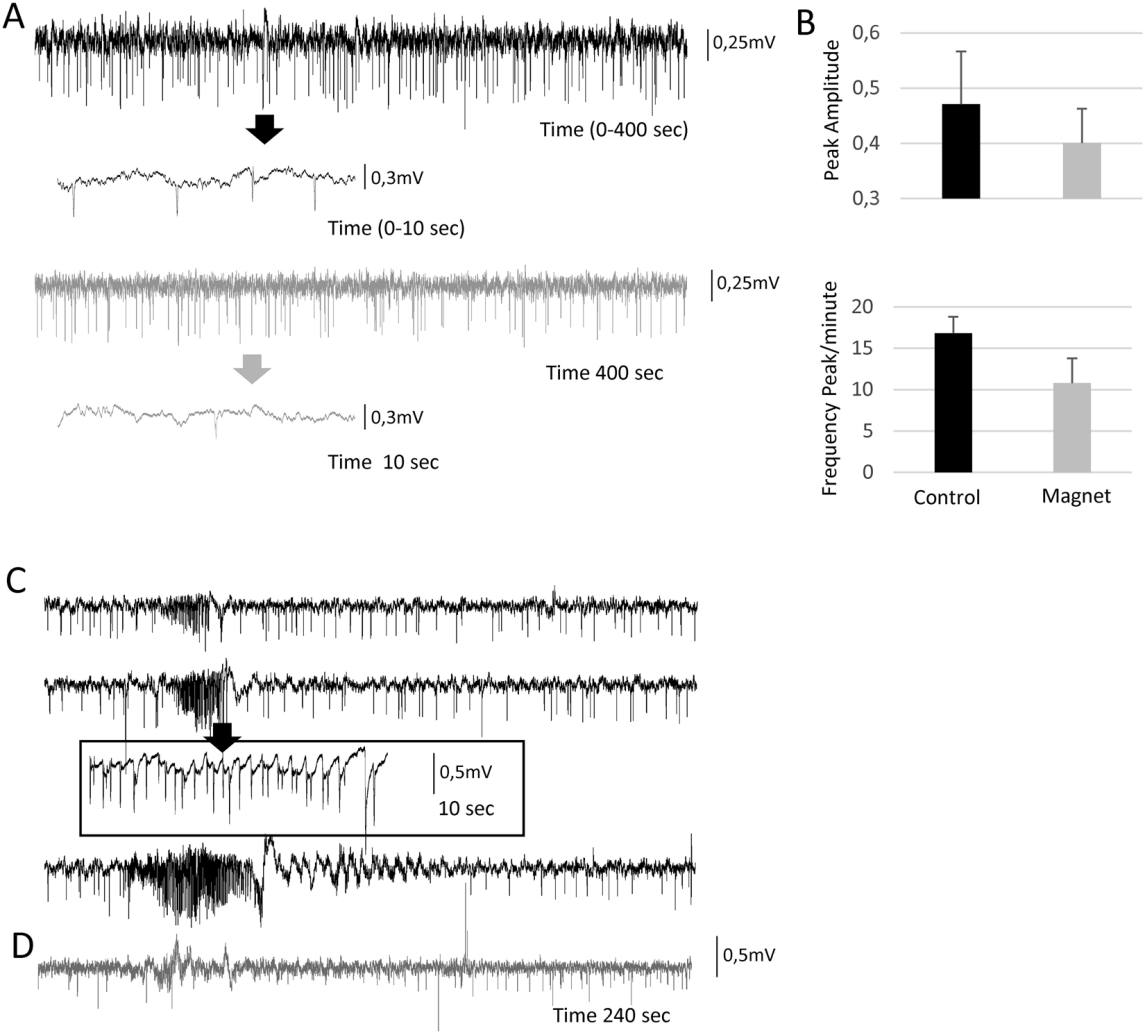
Static magnetic fields reduce the intensity and frequency of spontaneous seizures in the anaesthetized primate. (**A**) Spontaneous activity recorded in the monkey visual cortex in control (black) and with the magnet (grey) during non-seizure periods (400 seconds per recording). Note in the expanded traces the appearance of epileptic spikes (arrows). (**B**) Bar histogram representing Spike amplitude and frequency in control and after Magnet application. (**C**) Three different raw spontaneous traces that show the appearance of epileptiform activity under control conditions (3 seizures in 1 hour). (**D**) Recording fragment that shows the only epileptiform spontaneous activity recorded in the next hour once the magnet was placed close to the recording electrode.

Figure 5A shows two representative spontaneous recordings in the control situation (black) and during magnet stimulation (grey). Insets show an expanded 10-second view. In the control conditions, there is a continuous spiking activity, and a seizure is occasionally recorded (3 times in one hour, see below). The magnet decreased the appearances of the spontaneous spikes from 16,8 ± 2.01 to 10,8 ± 3.1 per minute (36% reduction), as well as their amplitude by 16% from 0.47 ± 0.096 to 0.4 ± 0.062 (p < 0.01 t-test) (Fig. 5B). With regard to the epileptic seizures, the 3 episodes spontaneously recorded during the first hour are shown in Fig. 5C (black) with an extended view of one episode in the inset (10 sec). During the first hour immediately after the magnet, we recorded only one small seizure-like activity (grey) that did not evolve (Fig. 5D).

#### Static magnetic fields reduce visual induced epileptiform activity

**Figure 6:**
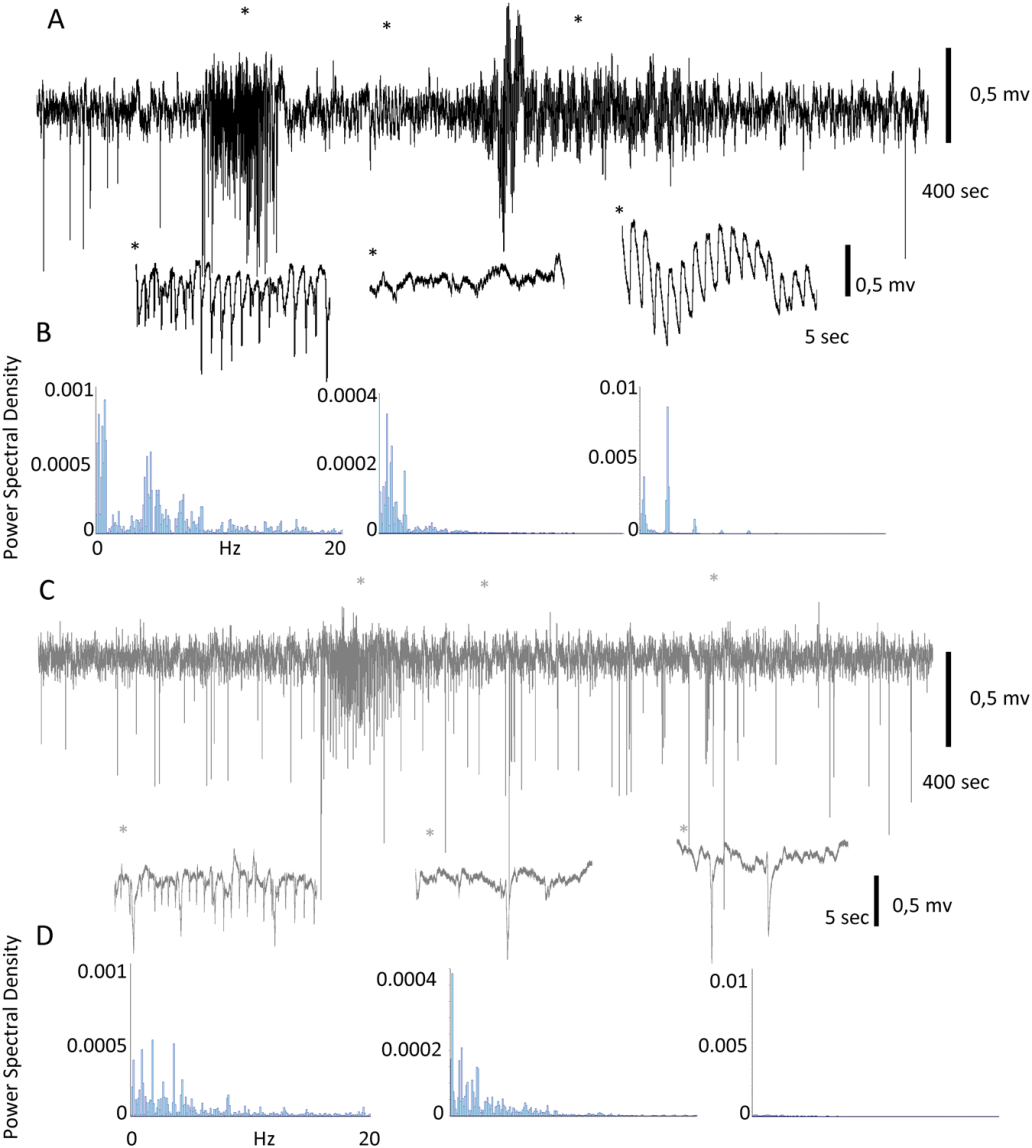
Magnet reduces sensory evoked seizures in the primate. (**A**) Visual evoked seizure recorded in V1 under control conditions. Areas marked with * are expanded below. (**B**) Power spectrum density analysis of the 3 epileptiform elements expanded in A (note the different scales on the Y axis). (**C**) Recording of the longest seizure evoked in the presence of the magnet (high-frequency stimulation was necessary to evoke it). In this case only, the initial paroxysmal activity (with a lower amplitude) is present. (**D**) Power spectrum density analysis of the 3 elements expanded in C (same scale as in A is used to enable direct comparisons).

In the visually induced seizure (Fig. 6A), a characteristic pattern emerges. Only 4 flashes at 2 Hz triggered the paroxysmal activity, which lasted for approximately 30 sec, followed by approximately 60 sec of low activity that finished in a strong oscillation before returning to baseline. Below the recording is an extended view of each phase of the seizure (10 sec corresponding to the *), as well as a power spectrum analysis of the segment; note the different scales in the PSD graphs.

After placing the magnet on the visual cortex, the situation substantially changed (Fig. 6B). In this case, the number of stimuli necessary for triggering the seizure was higher. Furthermore, in some cases, it was not possible to generate a seizure even with higher stimulation frequencies and longer times. Once triggered, the seizure exhibited a lower intensity, and only the first component (paroxysmal activity) was evident.

## Discussion

The main findings shown in this manuscript are two-fold: First, our data reinforce the view that SMF decrease cortical excitability. Second, we demonstrate that the application of SMF at a moderate intensity (0.5 T) can delay the appearance of the electrocorticographic characteristics of epilepsy in a rat pharmacological model and reduce the intensity and frequency of spontaneous and sensory-triggered seizures in an anaesthetized monkey with cortical damage, potentially due to continuous and long-lasting microelectrode penetrations for neural recording. Taken together, our results pave the way for the potential future use of static magnets in the treatment of epilepsy.

We are confident that our results are due to an effect of the magnetic field on neuronal activity and not to an artefact on the recording system. There are several supporting arguments: First, the screws are not ferromagnetic and are thus not affected by the magnetic field. Second, other elements of the system (amplifier/filters) were far away from the magnet and thus outside of the magnetic field influence. Third, previous studies have shown that static magnetic fields decrease cortical excitability without affecting the recording systems^10,11,13,15^.

Perhaps the most notorious characteristic of the effect shown here is its robustness. As shown in Figs 1, 2 and 5, the raw data clearly illustrate the difference between the sham and real SMF, which are also apparent by visual inspection.

Our data extend (and strengthen) previous promising findings obtained by other groups but, perhaps, are not as strong^4,5^. In the experiments of McLean *et al*., black Swiss mice received SMF for a period before audiogenic epileptic attacks were initiated. In addition to small improvements derived from the SMF, the main benefits were observed when SMF were applied in combination with pharmacological treatments, such as phenytoin. Given that SMF-effects require time to fully develop and last for minutes once the magnet is removed, we believe that removal of the SMF before the seizure was triggered would have attenuated the protective effect^11,12^. Moreover, subthreshold effects likely last for longer periods, thus facilitating pharmacological action. In our experiments, the magnet exerted its influence before and during the entire seizure; therefore, a stronger effect could be expected, and it appears to have occurred. However, it is worthwhile to consider that the protective effect of the magnet might have been even greater if combined with antiepileptic drugs. This interesting possibility that might open the door to SMF as adjuvant in the treatment of epilepsy should be investigated in future experiments. It is also interesting that while some anesthetics also have antiepileptic properties, this is not the case of sevoflurane, which can induce epileptiform activity^16–18^. In previous experiments in our lab involving normal animals, we did not identify EEG abnormalities, perhaps because we typically use sevoflurane levels below 5%. However, we cannot completely discard that sevoflurane could facilitate the Pilocarpine action in the rat model or contribute to the generation of the EEG abnormalities recorded in the primate. In any case, our main finding stands and is reinforced because the magnet was able to reduce the seizure-like events observed in both animal models.

The results obtained with sensory stimulation are particularly interesting. It is well known that epilepsy increases cortical excitability, which could ultimately cause permanent damage that abolishes physiological sensory responses. Our results clearly demonstrate that SMF application diminishes cortical excitability and stably maintains the responses to peripheral stimulation, thus preserving (at least for a period of time) the functional integrity of the circuit. In previous experiments, we demonstrated that SMF reduced sensory responses in the visual cortex, thereby increasing the threshold for visual detection and decreasing the firing rate of individual neurons but maintaining the specific visual properties of the neurons (i.e., direction and orientation selectivity)^12^. In the current experiments, sensory evoked potentials obtained during SMF were maintained very close to the control potentials, likely because the magnet counterbalanced the effect of the Pilocarpine without going further. Moreover, recent experiments have shown that the application of SMF over the visual cortex reduces the discomfort in an experimental model of photophobia in humans^15^, which again supports the idea that SMF attenuate cortical hyperexcitability.

With regard to the mechanisms that underlie the effects produced by SMF, to date, there is no definitive explanation. It has been shown that static magnetic fields of moderate intensity (0.12 T) can decrease the release of Ach in the mouse neuromuscular junction^19^. The change in calcium influx at the presynaptic terminal has been the proposed mechanism to explain this effect^20,21^. Furthermore, SMF appear to mimic the action of nifedipine (an inhibitor of calcium channels) and reverse the effects produced by Bay K8644, an L-type Ca^2+^ activator^22^. In these cases, the hypothesis is that SMF reorient membrane phospholipids and deform the calcium channels, which interferes with the membrane permeability to that ion.

This mechanism is compatible with the long times that are necessary to obtain the SMF effects (minutes) and the recovery periods observed in previous experiments^11,12^. It may also be compatible with the effect described in this manuscript because a decrease in calcium influx would decrease cortical excitability, globally depressing synaptic activity. Interestingly, calcium is an intracellular mediator that is involved in a myriad of cellular processes; therefore, changing the calcium influx could trigger other subtle long-term effects, which are not evident in our preparation. Furthermore, it has been proposed that SMF can influence cellular growth and the size of the cytoskeleton^23^. This proposal is not likely to be the mechanism of the effects described in our experiments due to the longer time scales required for these processes. For the chronic treatments necessary for epilepsy therapy, it is interesting that repetitive daily exposure to static magnetic fields in the range used in this work decreases in the expression of MAP-2 and prevents some of the toxic effects of MK801 by modulating the expression of particular NMDA receptor subunits^24^. Interestingly, *in vitro* SMF are able to decrease NMDA mediated plasticity in hippocampal neurons^25^. A decrease of NMDA receptor mediated activity would be a strong candidate behind a global drop in cortical excitability, as well as an increase of potassium conductance. Unluckily, from our experiments, we can only speculate the specific mechanisms, alone or in conjunction, that may be mediating the SMF action in our models. Our results open the door to new experiments *in vitro* specifically designed for this purpose.

It has also been suggested that SMF could change the ionic distribution across the membranes, thus modifying the membrane potential and producing an instantaneous effect; moreover, with the use of very strong fields (24 T)^26^, SMF can modify the axonal speed conduction. None of these aspects appear to be applicable to our data because the effects require several minutes to develop and the latency of the somatosensory evoked potentials did not change with magnetic stimulation.

If, as indicated, calcium is involved in a complex regulatory process that is responsible, at least in part, for the effects of static magnetic stimulation, it is tempting to speculate that astrocytes could be part of this equation. Astrocytes, the most abundant glial cells, operate through the so-called tripartite synapses and are involved in the regulation of many physiological parameters at the level of the glia-neuron interface, including neurotransmitter release and blood flow. Astrocyte excitability is based on the intracellular calcium levels (refer to Perea *et al*.^27^. for a review); therefore, a change in the intracellular calcium would completely alter the homeostasis of the cortex, in this case in a positive (protective) way. Modulation of astrocyte activity^28^ would be compatible with the time periods necessary to implement the SMF effects.

Although the path to the possible use of SMF as an antiepileptic therapy in humans remains far, it could be useful with respect to numerous considerations regarding practical issues, namely, how it could be administered and the safety limits. Due to the physical characteristics of the magnets used in this study, the most parsimonious application would be to locate the magnet directly on the epileptic focus. This step can be easily accomplished using special helmets, such as those designed for Parkinson’s disease studies (Neurek S.L. Toledo, Spain). For chronic treatments (i.e., 1 hour per day for 30 days) that attempt to achieve a long-lasting effect, similar to the rTMS protocols used for epilepsy^5,18^ or other mental illnesses^19^, safety concerns should be required. Continuous application of the magnet has been tested for periods of up to 2 hours^29^, without detectable changes in neuron-specific enolase (NSE) and protein S-100, which are sensitive markers for neural and glial brain damage, respectively. Moreover, there was no sign of cognitive dysfunction. Furthermore, in experiments that involved behaving monkeys in which the magnet was applied to the visual cortex for several days (daily sessions of 4 hours/day^12^), no neurological damage was identified. These numbers are within the limits established by the World Health Organization (200 mT during the working day) and also would accomplish the safety guidelines established for the use of MRI equipment^30^. Thus, 1 hour per day would be well inside the safety limits, and we hypothesize that application for a number of consecutive days could trigger more permanent changes in the altered brain-network configuration, similar to those achieved by rTMS protocols.

In summary, we described a clear effect of SMF, which can delay the appearance and reduce the intensity of epileptiform activity in two different species, rat and monkey. Although we are fully aware that many questions should be answered and that human studies should be performed, we believe that our results set the basis for future research in the treatment of refractory epilepsy using static magnets (as a non-invasive neuromodulatory technique), perhaps as an adjuvant strategy for pharmacological interventions.

## Methods

All procedures followed the rules of the Physiological Spanish Society, the International Council for Laboratory Animal Science, and the European Union (No. 2010/63/EU) and were approved by the ethics committee for animal research of the University Hospital of A Coruña.

### Experiment 1 (rat)

#### Animals

Fourteen Sprague-Dawley male rats were used in this work. Seven rats were classified as Magnet and 7 rats were sham (see below). The experiments were carried out in such a way that two animals were always recorded simultaneously, one “magnet” (real stimulation) and the other “sham” (placebo stimulation).

#### Stereotactic implantation of recording electrodes

Animals were placed in a cage for anesthesia induction by applying 5% sevoflurane. Once the hindpaw and corneal reflexes disappeared, the animals were placed in a stereotaxic frame to implant stainless screws (1.2-mm diameter, 4-mm length). A small guide hole was created using a hand drill; we then started to screw lowering the screws through the bone without touching the cortex. This provided an excellent and stable signal to noise ratio and avoided potential movement^31^. One screw as an active electrode was placed on the area of the somatosensory cortex that corresponds to the hindpaw coordinates (AP: −1.5; ML 2)^32^, and the second screw as a reference electrode was placed in the contralateral Visual cortex (AP: −6.3; ML: 3.5)^32^. Once the screws were placed, a drop of instant glue was applied to avoid small movements.

#### EEG recordings

A differential amplifier (Model 1700 A-M System, LLC, Calsborg, WA, USA) was used to continuously record the EEG signal. The signal was amplified (x1000) and filtered (1 Hz–500 Hz). The signal was digitized at 20 KHz by a 1401 CED A/D convertor card (Cambridge Electronic Design, UK) and stored using Spike 2 software (Cambridge Electronic Design, UK) in a PC for online checking and posterior analysis. The anesthesia level was fixed at 1.5–2% of sevoflurane to maintain stable slow-wave activity during the control conditions (the initial period without the induction of epilepsy). The animals remained anaesthetized throughout the experiment.

#### Sensory Stimulation

Electrical stimulation was applied using a GRASS S88 Stimulator and a Grass PSIU6X Photoelectric isolator (Grass Instruments, Quincy, MA, USA) for bipolar stimulation. Bipolar needle electrodes for stimulation were subcutaneously placed at the wrist on the contralateral hind paw to obtain somatosensory evoked potentials that enabled us to determine the integrity of the sensory responses in the different experimental conditions. The stimulation parameters were 50 stimuli at 0.2 Hz frequency, 0.5 ms pulse duration. The intensity was adjusted in the control conditions as the intensity that evoked a response at 80% of the stimuli. In all experiments, it was between 0.8 and 1 mA. The stimulation protocol was applied every 30 min during the entire experiment.

#### Drug administration

We used the lithium-Pilocarpine model to induce epilepsy^33^. For each experimental session, two animals were injected (i.p.) with LiCl (127 mg/kg) the day before the actual experiment and were subsequently returned to the cage. The day of the experiment, the two animals were anaesthetized (see above), and the EEG quality was verified; the level of anesthesia was adjusted to obtain a stable slow-wave state with clear “up” and “down” states (refer to Figs 1 and 2). Once an initial control (spontaneous and evoked responses) was obtained, Scopolamine (1 mg/kg) was injected (i.p.). After thirty minutes, two doses of Pilocarpine (20 mg/kg each), 30 minutes apart, were administered (i.p.).

#### Application of moderate static magnetic fields (SMF)

Prior to the first Pilocarpine dose, we determined which animal would receive real stimulation (magnet) and which one would receive sham stimulation by tossing a coin. Thus, during each experimental session, one animal from each pair was exposed to a real magnetic stimulation and the other animals received a placebo stimulation. We placed the real magnet on the “*magnet animal”* and the replica on the *“sham animal”*. The magnet was placed with the field lines perpendicular to the cortical surface. We used a cylindrical nickel-plated (Ni–Cu–Ni) NdFeB magnet with a 45-mm diameter and a 30-mm thickness that weighed 360 g (NEUREK S.L., Toledo Spain). The maximum amount of magnetic energy stored in this magnet was 45 MGOe, with a nominal strength of 765 N (78 kg). Our own measurements^34^ showed a magnetic field of 0.5 T next to the magnet and ∼0.3 T at 1 cm. A nonmagnetic replica of identical appearance and weight (i.e., indistinguishable from the magnet) was used for the sham stimulation (NEUREK S.L., Toledo Spain).

#### Prolonged EEG monitoring

Recordings from the two animals were continuously obtained for up to 6 hours. From the beginning of the experiment, a sensory stimulation protocol (50 stimuli at 0.2 Hz frequency, 0.5 ms pulse duration, and 0.8–1 mA intensity) was administered every 30 minutes.

### Experiment 2 (monkey)

#### Animals

One anaesthetized (sevoflurane 1–3%) male macaque monkey (Macaca mulatta) was used for this experimental block.

#### Recording

The animal was placed in a stereotaxic frame. A craniotomy was opened over the visual cortex, overlying and posterior to the lunate sulcus, and a tungsten electrode (10 MΩ) was introduced in the primary visual cortex for electrocorticogram recording. A second craniotomy was opened above the visual thalamus, and a second electrode was downloaded to the Lateral Geniculate Nucleus (LGN) for extracellular recording. The cortical and thalamic activities were recorded using a differential amplifier (Model 1700, A-M System LLC, Calsborg, WA, USA), and both signals were amplified (x1000) and filtered (0.5 Hz-3000 Hz) to have local field potentials and multiunit activity. Recordings were digitized by an A/D convertor card (1401 CED, Cambridge Electronic Desing, UK) and stored using Spike 2 software (Cambridge Electronic Desing, UK) in a PC for online checking and posterior analysis.

#### Seizure like events

The original purpose of this experiment was to assess the contribution of feedback connections on thalamic activity under different conditions. However, after several sessions of continuous recordings, we detected the presence of epileptiform activity in the recording electrode at V1, characterized by intermittent episodes of spike-wave activity (clearly visible by visual inspection), spontaneous in some cases, but mainly triggered by photic stimulation. We hypothesized that the abnormal activity was due to the trauma of repeated penetrations in this area of the cortex. These episodes were easily induced by visual stimulation: Visual stimuli were flashes at 2 or 4 Hz generated with a stroboscope until the appearance of the seizure (longest stimulation time was 1 minute). To analyze these EEG abnormalities, for each raw recording, the Hilbert transform of the signal was calculated. The envelope of the transform was subsequently performed and smoothed. Finally, using an adaptive threshold computed as the ratio between the signal noise and activity level, the abnormal activity in the EEG was detected and processed (see below).

#### Application of moderate static magnetic fields (SMF)

After characterizing these EEG abnormalities (amplitude, duration, and frequency), we placed a magnet identical to the one previously described in the vicinity of the electrode and re-checked the EEG characteristics again at different time intervals. Because of the presence of the recording electrode, the magnet was rotated and slightly separated from the cortical surface. Recordings were extended over eight hours.

#### EEG data analysis

Data analyses were performed with Spike2 software (Cambridge Electronic Design, v8.03) and home-made routines in MATLAB.

The raw EEG signal was downsampled to 1 KHz, and an IIR Butterworth-like filter between 1 and 70 Hz was applied. Every 10 minutes, an epoch of 30 seconds was automatically selected, and each epoch was analyzed in terms of the root mean square (RMS) and power spectral density (PSD) from 1 to 70 Hz and classified as Delta (1–4 Hz), Theta (4-8Hz), Alpha (8–13Hz), Beta (13–30Hz) and Gamma (30–70Hz).

RMS was used as an estimator of the EEG amplitude. For a given set of values, RMS is the square root of the arithmetic mean of the squares of these values. In the case of a DC signal or a non-sinusoidal signal, RMS provides an estimation of the averaged amplitude. To calculate the RMS, we used the specific tools included in the spike2 software.

Values are expressed as the mean ± standard error. Due to the small population sample, the Mann-Whitney U test for unrelated samples was used to compare the RMS values and PSD at the different frequency bands during the different experimental conditions. All results were considered significant at P < 0.05.

To perform the analysis of the evoked somatosensory responses, the recorded signal was preprocessed. The raw signal was downsampled from 40 kHz to 10 kHz, and a Savitzky-Golay filter was applied for smoothing purposes. To increase the signal-noise ratio, the signal was subsequently fitted by a polynomial function. The evoked potential was averaged for all trials, and we calculated the peak to peak amplitude and the latency of the second peak for all animals and conditions.

To identify significant differences, a paired sample t-test was employed. The results were considered significant at P < 0.05.

## Electronic supplementary material

Supplementary tables
