# Supplementary material for: Static magnetic fields reduce epileptiform activity in anesthetized rat and monkey
Source: Sci Rep. 2018 Oct 30;8:15985. doi: 10.1038/s41598-018-33808-x (PMC6207659; doi:10.1038/s41598-018-33808-x)
Supplement: Supplementary file 1 — Supplementary tables [file 41598_2018_33808_MOESM1_ESM.pdf]

Static magnetic fields reduce epileptiform activity in anesthetized rat and monkey

Rivadulla Casto, Aguilar Juan, Coletti Marcos, Aguila Jordi, Prieto Sandra, Cudeiro Javier

**Time from Pilocarpine injection (minutes)**	**PowerV²/Hz. 1-8 Hz** Sham (Mean)	SEM	**PowerV²/Hz. 1-8 Hz** Magnet (Mean)	SEM
-45	0,0032	0,0011	0,0018	0,0008
-15	0,0036	0,0013	0,00165	0,0009
15	0,00645	0,0029	0,00215	0,0004
45	0,0166	0,0033	0,0032	0,0016
90	0,01225	0,0021	0,0032	0,0009
150	0,009	0,00098	0,0045	0,00087

Supp table 1: numerical values of data represented in figure 3A

Static magnetic fields reduce epileptiform activity in anesthetized rat and monkey

Rivadulla Casto, Aguilar Juan, Coletti Marcos, Aguila Jordi, Prieto Sandra, Cudeiro Javier

**Time from Pilocarpine injection (minutes)**	**% of change in control EEG Root Mean Square** Sham (Mean)	SEM	**% of change in control EEG Root Mean Square** Magnet (Mean)	SEM
-60	-4,03729205	1,65068692	0,62709489	2,96269356
-50	2,23885664	1,1294745	-0,38274535	2,0759358
-40	1,79843542	0,99496995	-0,24434955	2,60540141
-30	4,52743077	1,10412075	-4,15867978	4,36972379
-20	7,60484235	2,95814399	-4,87342466	5,82760531
-10	8,67847118	1,67056896	3,77170182	4,316097
0	7,79175057	2,93722118	-3,65172133	5,09574671
10	27,7172273	6,84459423	-3,33233888	4,0331728
20	16,7922179	3,00693104	-5,15076216	5,37532509
30	25,6384785	6,73117495	6,15870996	6,04030913
40	52,7555531	13,4091995	14,0661574	6,34920507
50	89,4747714	22,6847556	30,4209819	11,7188908
60	57,6960164	7,39938557	34,4794224	9,03799925
70	63,5622565	6,36900788	33,2676614	8,92587073
80	71,7597997	10,8169411	38,0332149	8,25732835
90	59,6137067	6,94480474	52,4485474	12,491417
100	52,3878403	4,70226119	56,360198	10,7778127
110	57,5319895	5,57241807	59,3285127	13,1641269
120	60,8644327	4,9281708	59,5216672	9,7923924
130	51,5629974	5,7220324	60,1455757	11,5722356
140	49,889291	6,72149266	71,4714056	9,04599961
150	46,166058	7,73808863	57,8021675	12,6606453

160	46,6552999	7,33055501	61,833101	10,1210298
170	54,5341508	6,53663175	42,6379906	17,3394381
180	53,094101	9,11108537	60,2356422	17,5027502
190	46,4538748	8,12226108	58,4653603	11,3765829
200	36,2635323	9,85033515	42,3545967	15,4771883
210	32,0883203	12,9677919	40,6349769	24,3607722
220	36,6387374	12,2248234	47,9285506	15,7656654
230	48,8624777	8,43251895	50,027876	14,0169465
240	23,9569205	11,6782371	38,751728	24,2763264
250	51,1290613	8,34463754	70,2683912	16,2662339
260	54,4080337	7,74947904	59,8305322	12,6742682
270	47,9262517	7,29395892	66,7281657	14,7672515
280	46,2713818	6,1763316	61,8273496	18,5801858

Supp table 2: numerical values of data represented in figure 3B

Static magnetic fields reduce epileptiform activity in anesthetized rat and monkey

Rivadulla Casto, Aguilar Juan, Coletti Marcos, Aguila Jordi, Prieto Sandra, Cudeiro Javier

Time (min) from first Pilocarpine injection	% of change in peak to peak amplitude (Mean) SHAM	SEM	% of change in peak to peak amplitude (Mean) MAGNET	SEM
-60	1	0	1	0
-30	0,79669	0,051	0,79669	0,0276
0	0,816165	0,030	0,871908	0,0312
30	1,419978	0,096	1,194406	0,0446
60	2,293282	0,208	1,180213	0,0520
90	2,176007	0,194	1,214168	0,062461
120	1,494477	0,100	1,199679	0,0582
150	1,041988	0,086	1,239624	0,0945

Supp table 3: numerical values of data represented in figure 4B
